# The impact of nutritional intervention and resistance training on muscle strength and mass in healthy older adults—a comparative analysis

**DOI:** 10.3389/fnut.2025.1640858

**Published:** 2025-08-18

**Authors:** Yongye Ma, Ruixiang Yan, Yueming Li, Duanying Li, Xiaoning Sun, Tao Chen, Xingyu Liu

**Affiliations:** ^1^School of Athletic Training, Guangzhou Sport University, Guangzhou, Guangdong, China; ^2^Badminton Technical and Tactical Analysis and Diagnostic Laboratory, National Academy of Badminton, Guangzhou Sport University, Guangzhou, Guangdong, China

**Keywords:** nutritional intervention, resistance training, muscle strength, muscle mass, healthy older adults

## Abstract

**Objective:**

A growing body of evidence confirms that nutritional supplementation strategies combined with resistance training can enhance muscle strength and mass in older adults. However, the optimal supplementation approach remains unclear. This study aimed to evaluate the comparative efficacy of different nutritional interventions combined with resistance training on muscle strength and mass in healthy older adults and determine the optimal strategy.

**Methods:**

A systematic search was performed across three major biomedical databases (PubMed, Web of Science, and EMbase) to identify randomized controlled trials (RCTs) investigating the effects of nutritional supplementation combined with resistance training on muscle strength and mass in healthy older adults. A total of 19 eligible RCTs were included. The search covered literature from database inception to April 2025. Two researchers independently screened studies against predefined eligibility criteria and assessed methodological quality using the Cochrane risk-of-bias tool. Stata 18.0 was used to conduct network meta-analysis.

**Conclusion:**

Compared with resistance training alone, protein supplementation combined with resistance training significantly enhanced muscle strength [Standardized Mean Difference (SMD) = 0.45, 95% confidence interval (CI): 0.20,0.69; surface under the cumulative ranking curve (SUCRA) = 98.7%] and muscle mass [Mean Difference (MD) = 0.37, 95%CI: 0.04,0.70],whereas creatine supplementation demonstrated non-significant effects on muscle strength versus training alone (SMD = 0.03, 95% CI: −0.35,0.42) but yielded the most pronounced improvement in muscle mass (MD = 2.18, 95%CI: 0.92,3.44; SUCRA = 99.9%), outperforming both protein and β-hydroxy-β-methylbutyrate (HMB) interventions, with HMB supplementation critically failing to demonstrate significant benefits for muscle strength (SMD = −0.22, 95%CI: −0.57,0.12) or mass outcomes (MD = 0.05, 95%CI: −0.33,0.44).

**Systematic review registration:**

https://www.crd.york.ac.uk/PROSPERO/view/CRD420251026016.

## 1 Introduction

With the accelerating pace of global population aging, the World Health Organization predicts that the proportion of individuals aged 65 and older will reach 16% by 2050 (1). This demographic shift has positioned age-related sarcopenia as a major public health challenge. Recent epidemiological data indicate that the prevalence of sarcopenia among community-dwelling older adults aged 60 and above ranges from 10 to 27% worldwide (2). Sarcopenia is associated with increased risks of falls (OR = 3.21), disability (HR = 1.79), and all-cause mortality (RR = 1.58) (3).

The degenerative loss of muscle mass and function, a hallmark of sarcopenia, is closely linked to age-related anabolic resistance (4). Resistance training serves as a critical intervention to mitigate muscle decline. The American College of Sports Medicine (ACSM) recommends that older adults engage in systematic resistance training ≥2 times per week at 60–80% of 1-repetition maximum (1RM) intensity for 8–12 weeks to significantly enhance muscle strength and lean body mass (5). Beyond exercise interventions, various nutritional supplements have emerged as adjunct strategies to counteract muscle atrophy, including whey protein, creatine, and β-hydroxy-β-methylbutyrate (HMB). These supplements act through distinct physiological pathways: whey protein provides essential amino acids (particularly leucine) to activate the mTORC1 pathway, thereby stimulating muscle protein synthesis (6); creatine enhances phosphocreatine reserves to improve type II muscle fiber recruitment (7); and HMB inhibits the ubiquitin-proteasome system to reduce muscle breakdown (8). While these mechanisms suggest potential benefits, their efficacy in practice is often constrained by factors such as insufficient nutritional support and inadequate training stimulus. For instance, when protein intake falls below 0.8 g/kg/day, the efficiency of resistance training-induced protein synthesis may decrease by 42% (9). Furthermore, even with adequate protein supplementation, up to 56% of amino acids may remain underutilized in the absence of mechanical loading (10). Thus, combined interventions integrating nutritional supplementation with resistance training are considered synergistic, particularly in older populations.

Although multiple randomized controlled trials (RCTs) have validated the effectiveness of resistance training combined with various nutritional strategies, existing evidence has limitations. Previous studies predominantly focus on evaluating single-nutrient interventions combined with resistance training, lacking direct or indirect comparisons among multiple nutritional approaches (11). Additionally, age-related metabolic alterations may modify dose–response relationships for nutritional supplements in older adults, warranting further investigation (12).

Network Meta-Analysis (NMA), an advanced evidence synthesis methodology, enables the integration of direct and indirect comparative evidence to rank multiple interventions quantitatively (13). This study is the first to employ NMA to systematically compare the effects of three mainstream nutritional strategies—protein, creatine, and HMB—combined with resistance training on muscle strength and mass in healthy older adults. The findings aim to establish a hierarchy of relative efficacy among these supplements, providing high-level evidence to inform personalized exercise-nutrition prescriptions and offering critical clinical insights for delaying age-related muscle decline.

## 2 Methods

This study adhered to the Preferred Reporting Items for Systematic Reviews and Meta-Analyses (PRISMA) guidelines, specifically the PRISMA extension for Network Meta-Analysis (PRISMA-NMA) (14). The protocol was prospectively registered in PROSPERO (Registration No. CRD420251026016).

### 2.1 Search strategy and inclusion/exclusion criteria

Two researchers independently conducted a systematic search across three major biomedical databases (PubMed, Web of Science, and EMbase) from inception to April 2025. The search strategy utilized the following Boolean terms: (“nutritional supplements” OR “dietary supplements” OR “nutrients”) AND (“resistance training” OR “strength training” OR “resistance exercise” OR “strength exercise”) AND (“elderly” OR “older adults” OR “aged” OR “aging population”) AND (“muscle strength” OR “muscle mass” OR “strength performance” OR “muscle hypertrophy”).

#### 2.1.1 Inclusion criteria

(1) Randomized controlled trials (RCTs) with accessible full texts.
(3) Participants: Community-dwelling adults aged ≥60 years (15), free from major chronic diseases, with normal physical/cognitive function and mental health (16).
(3) Interventions: Detailed protocols for nutritional supplementation (type, dose, frequency) and resistance training (intensity, frequency, duration).
(4) Outcomes: At least one validated measure of muscle strength or mass.

#### 2.1.2 Exclusion criteria

(1) Insufficient intervention details.
(2) Incomplete baseline/post-intervention data.
(3) Outcomes unrelated to muscle strength/mass.

### 2.2 Study screening and data extraction

Search results were imported into EndNote X8 (Clarivate Analytics) for removal of duplicate records. Two reviewers independently screened titles, abstracts, and full texts against eligibility criteria.

Data extraction was performed independently by two reviewers using a standardized template, with discrepancies resolved through consultation with a senior investigator. Extracted data included:

1. Study characteristics: First author, publication year, country.
2. Participant demographics: Sample size, sex distribution, mean age.
3. Intervention details: Nutritional supplement type (e.g., whey protein, creatine), dosage, resistance training protocol (e.g., intensity, frequency), and duration.
4. Outcome metrics: Mean and standard deviation (SD) for muscle strength and mass pre-and post-intervention. Missing data were requested from corresponding authors via email.

### 2.3 Risk of bias assessment

Two researchers assessed the methodological quality of included studies using the Cochrane Risk of Bias Tool. The Cochrane tool consists of the following domains: random sequence generation, allocation concealment, blinding of participants and personnel, blinding of outcome assessment, incomplete outcome data reporting, selective reporting, and other biases. Each domain was categorized as low risk, high risk, or unclear risk of bias. The risk of bias assessment was visualized using RevMan 5.1. A study was classified as high risk if it demonstrated high risk of bias in two or more domains, as low risk if five or more domains were rated as low risk, and as moderate risk in all other cases (17).

### 2.4 Statistical analysis

The statistical analysis was performed using Stata 18.0 software. For studies investigating muscle strength outcomes, which involved continuous numerical data with heterogeneous assessment tools and measurement units, the standardized mean difference (SMD) with 95% confidence intervals (95% CI) was employed as the effect size metric using random-effects models. In contrast, mean difference (MD) with 95% CI was utilized to pool effect sizes for muscle mass outcomes under fixed-effect models, due to the consistency in measurement units across studies and low heterogeneity confirmed by the global inconsistency test (*p* = 0.789). Global inconsistency was assessed through the node-splitting approach. This model selection strategy follows the Cochrane Handbook recommendations for addressing clinical heterogeneity in network meta-analyses (18). The significance of efficacy differences in muscle strength and mass was examined using SMD and MD (95% CI), with statistical significance defined as *p* < 0.05. The efficacy ranking of nutritional interventions was determined by calculating the surface under the cumulative ranking curve (SUCRA). SUCRA values range from 0 to 100%, where higher values indicate greater probability of superior therapeutic effectiveness. Funnel plots were used to assess publication bias and small study effects in the NMA.

## 3 Results

### 3.1 Search results

**Figure 1:**
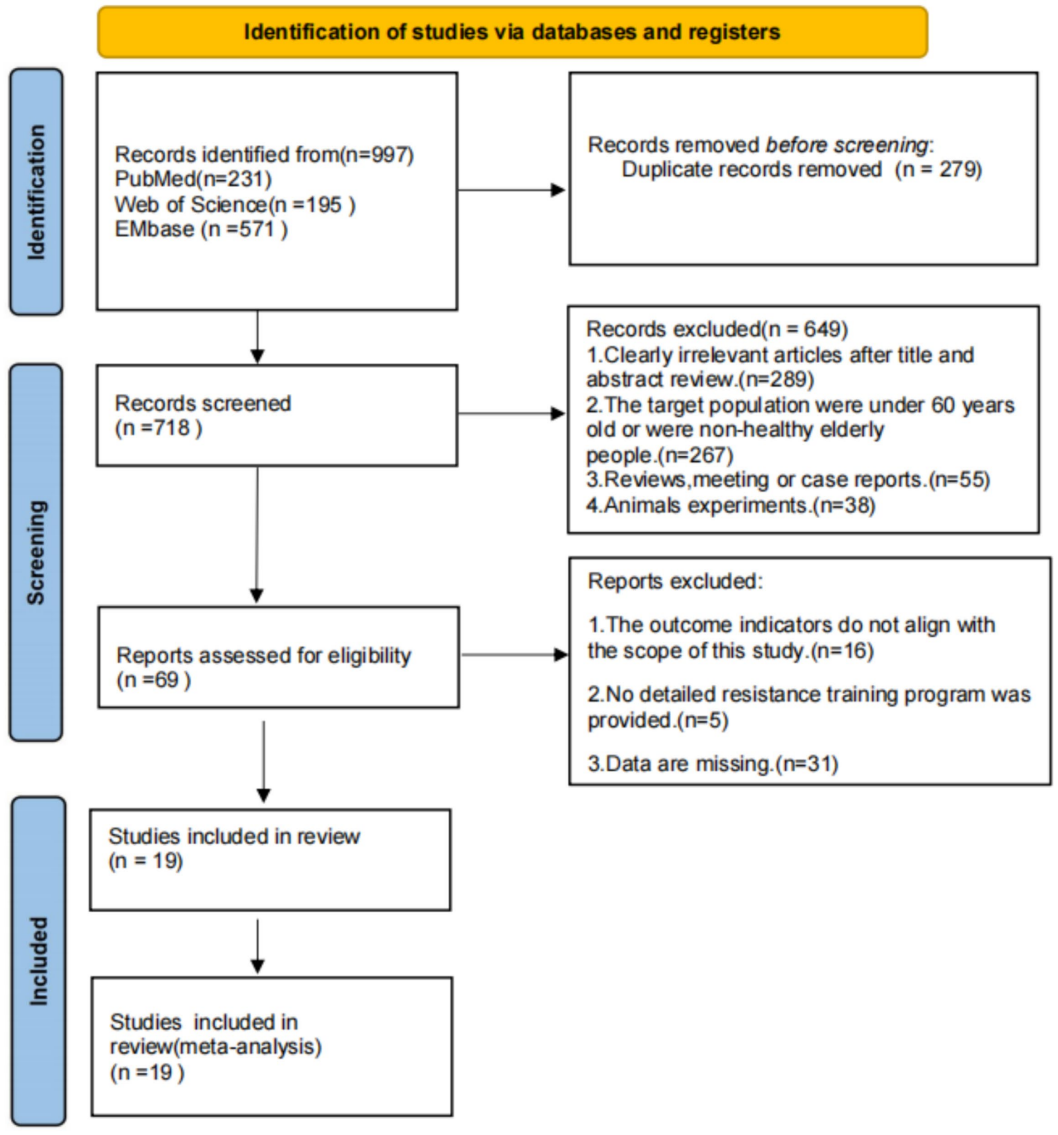
Literature screening flowchart.

A systematic search across PubMed, Web of Science, and EMbase yielded 997 records. After applying eligibility criteria, 19 randomized controlled trials (RCTs) were included in the network meta-analysis (NMA). The study selection process, adhering to PRISMA guidelines, is detailed in Figure 1.

### 3.2 Characteristics of included studies

The 19 studies in this network meta-analysis involved 997 healthy older adults and evaluated three interventions: 11 studies focused on protein supplementation, 5 on creatine supplementation, and 3 on HMB supplementation. Muscle strength outcomes were reported in 16 studies, while muscle mass outcomes were reported in 18 studies. The basic characteristics of the included studies are summarized in Table 1.

**Table 1:** Characteristics of included studies.

Author	Experimental group (*n*)	Control group (*n*)	Gender (M/F)	Age	Intervention duration	Nutritional supplement	Resistance training frequency	Outcome measures
Daly (41)	53	47	F	C73.6 ± 7.7 E72.1 ± 6.4	4 months	6 servings/week, 220 g lean red meat per serving	Twice a week, 45–60 min/session	LBM, Leg extension strength
Pinto (42)	13	14	Not reported	E67.4 ± 4.7 C67.1 ± 6.3	12 weeks	Creatine (5 g/day)	Three times a week, 60 min/session	LBM, 10RM leg press, 10RM bench press
Granic (43)	10	10	M12 F8	C70.8 ± 4.0 E72.0 ± 2.7	6 weeks	Post-exercise supplementation with 500 mL of 3.6% fat whole milk	Twice a week	Grip strength, ASMM
Chalé (44)	42	38	Not reported	C77.3 ± 3.9 E78.0 ± 4.0	6 months	Daily intake of 40 g whey protein concentrate	Three times a week	LBM, leg extension strength
Nakayama (45)	61	61	M30 F92	C70.4 ± 0.7 E71.4 ± 0.8	6 months	One bottle (200 mL) of acidified milk protein beverage per day	Once a day	LBM, grip strength, knee extension strength
Seino (46)	40	40	M13 F67	E73.4 ± 4.3 C73.7 ± 4.3	12 weeks	200 mL/day of milk protein	Twice a week, 60 min/session	LBM, grip strength, knee extension strength
Bernat (47)	12	12	M	C58.16 ± 5.85 E59.00 ± (7.07)	8 weeks	Creatine supplementation at 0.1 g/kg/day	Twice a week	Leg press, bench press
Kirk (48)	22	24	M21 F25	C66 ± 4 E69 ± 6	16 weeks	Whey protein administered 3 times daily (TID) at 1.5 g/kg/day (0.5 g/kg/meal)	Three times a week	SMM, grip strength, leg extension
Galbreath (49)	17	19	F	C66.0 ± 4.3 E65.5 ± 5.2	14 weeks	Protein intake of 1.2 g/kg/day	Three times a week,30 min/session	LBM, leg press
van Dongen (50)	82	86	M66 F102	C76.9 ± 6.5 E74.7 ± 5.8	12 weeks	25 g protein per meal (three meals daily)	Twice a week, 60 min/session	LBM, leg press, leg extension strength
Mori (51)	25	25	F	C70.6 ± 4.2 E70.6 ± 4.2	24 weeks	Whey protein at 1.2 g/kg/day	Twice a week	Grip strength, knee extension, upper/lower limb muscle mass
Uchida (52)	18	20	F	60–79	12 weeks	110 g chicken consumed three times weekly	Three times a week	Upper/lower limb and whole-body LBM
Brose (53)	14	14	M15 F13	E(F)68.7 ± 4.8 (M)70.8 ± 6.1 C(F)68.3 ± 3.2 (M)69.9 ± 5.6	14 weeks	5 g/day creatine monohydrate	Three times a week	LBM, grip strength, leg press, knee extension strength
Osuka (54)	36	38	F	C71.8 ± 4.1 E73.5 ± 4.2	12 weeks	1,500 mg/day Ca-HMB	Twice a week, 60 min/session	LBM, grip strength, knee extension strength
Stout (55)	24	24	M22 F26	C73 ± 1 E73 ± 1	24 weeks	3 g calcium-bound HMB (HMB-Ca) twice daily	Three times a week	LBM, grip strength, leg press, bench press, knee extension strength
Din (56)	8	8	F	C68.5 ± 1.0 E67.8 ± 1.1	6 weeks	3 g/day free acid β-hydroxy-β-methylbutyrate (HMB-FA)	Three times a week	Leg extension, thigh lean mass
Aguiar (57)	9	9	M	C66 ± 6 E64 ± 4	12 weeks	5 g creatine monohydrate once daily	Three times a week	LBM, SMM
Griffen (58)	9	9	F	C67 ± 1 E68 ± 1	12 weeks	25 g whey protein isolate twice daily	Twice a week	LBM, SMM
Chrusch (59)	16	14	F	C71.1 ± 1.8 E70.4 ± 1.6	12 weeks	Mean daily creatine dose:6.2 ± 0.3 g/day	Three times a week	LBM

Evaluation tool abbreviations: LBM, The Lean Body Mass; ASMM, The Appendicular Skeletal Muscle Mass; SMM, The Skeletal Muscle Mass.

### 3.3 Risk of bias assessment

**Figure 2:**
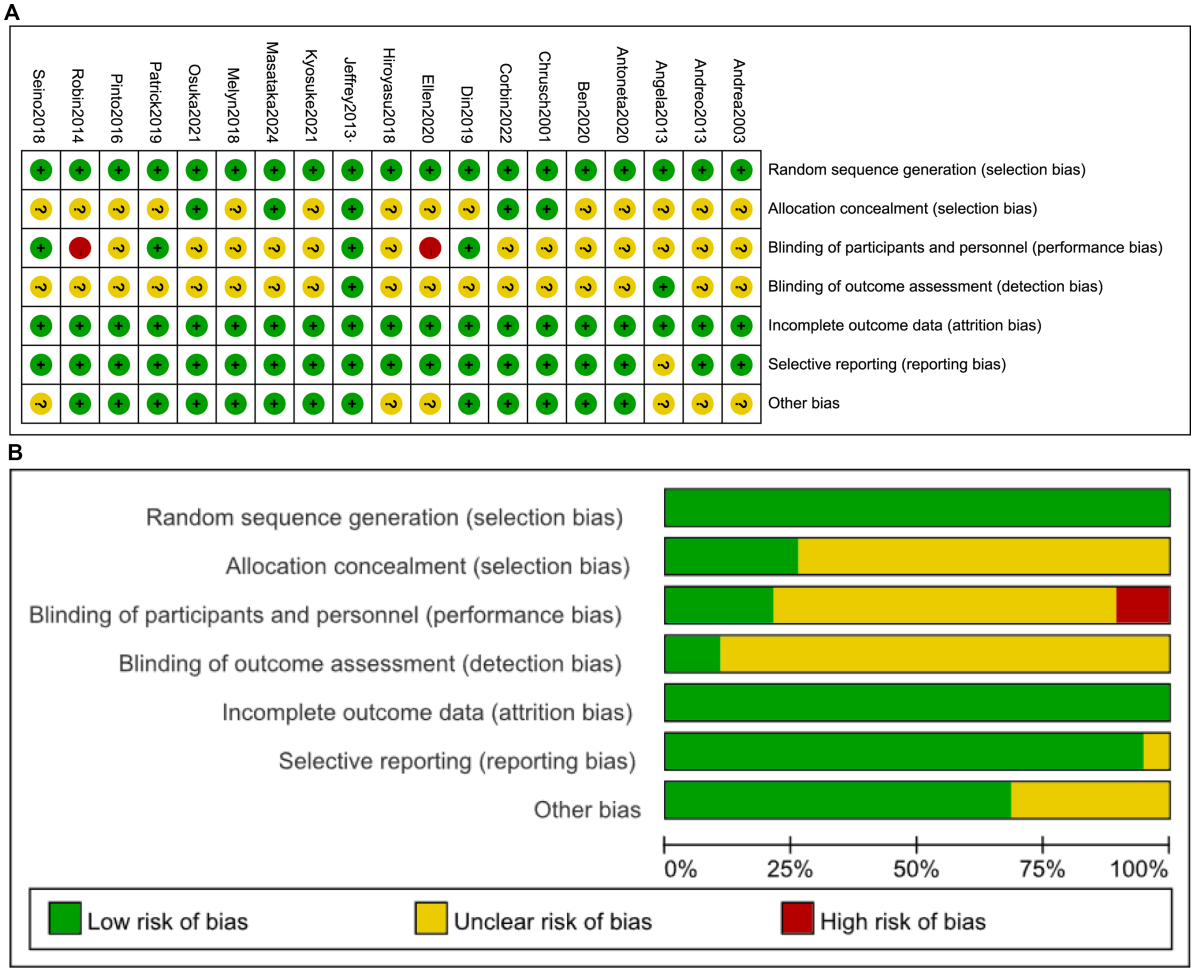
Risk of bias assessment: **(A)** across all studies; **(B)** per-item risk distribution within individual studies.

As illustrated in Figure 2, the assessment revealed domain-specific limitations frequently observed in sports nutrition RCTs. Specifically, allocation concealment demonstrated unclear risk in 73.7% of studies (14/19), while blinding of participants and personnel showed unclear risk in 68.4% (13/19). This pattern reflects the inherent complexities of blinding procedures and the frequent impracticality of complete blinding in nutritional interventions owing to supplement palatability or administration routes (19, 20). Performance bias was rated high risk in 10.5% of studies (2/19) due to unblinded researchers. Detection bias exhibited unclear risk in 89.5% of studies (17/19) owing to insufficient methodological details regarding muscle mass assessment techniques. None of the RCTs included in this network meta-analysis were classified as high risk (defined as ≥2 high-risk domains), while 31.6% (6/19) achieved low risk and 68.4% (13/19) moderate risk.

### 3.4 Network meta-analysis results

#### 3.4.1 Consistency check

Consistency tests for the network meta-analysis revealed no significant inconsistency among the included RCTs. For muscle strength outcomes in older adults (*p* = 0.2001) and muscle mass outcomes (*p* = 0.789), both *p*-values exceeded 0.05, indicating homogeneity across studies.

#### 3.4.2 Network geometry

**Figure 3:**
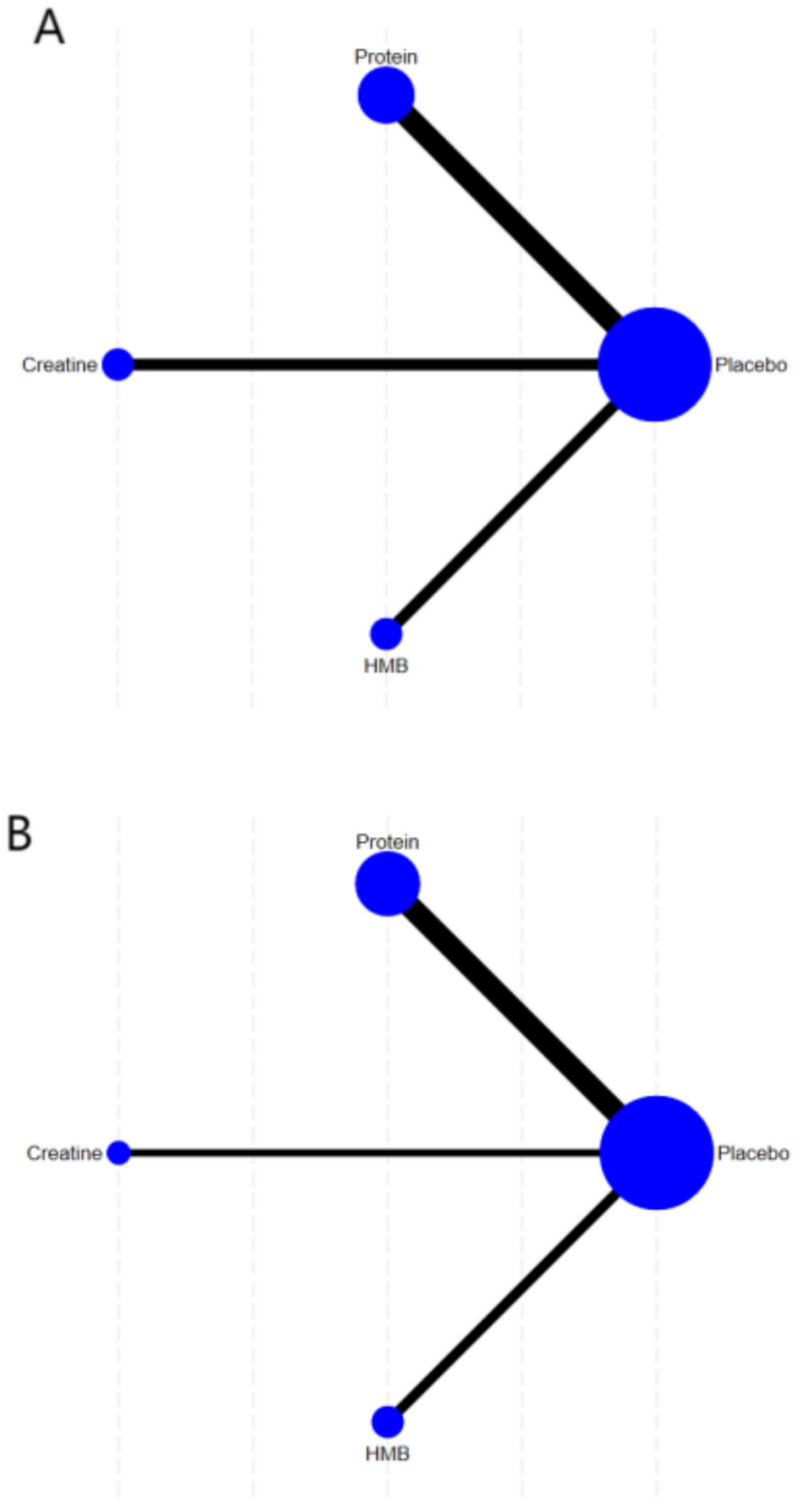
Network geometry of interventions for **(A)** muscle strength outcomes and **(B)** muscle mass outcomes.

The network geometry diagrams (Figures 3A,B) illustrate the comparative evidence structure of nutritional interventions for muscle strength and mass outcomes. Each node represents an intervention (placebo, protein, creatine, HMB), with node sizes proportional to the number of participants and line thickness reflecting the number of direct comparisons between interventions. The placebo node serves as the common comparator anchoring the network. For muscle strength outcomes (Figure 3A), protein supplementation demonstrates the most extensive direct evidence connections, while creatine shows predominant centrality in the muscle mass network (Figure 3B).

#### 3.4.3 Ranking probabilities

**Figure 4:**
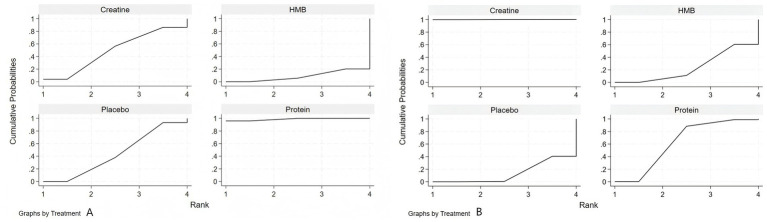
Surface under the cumulative ranking curve (SUCRA) for nutritional interventions on **(A)** muscle strength and **(B)** muscle mass in healthy older adults.

As shown in Figure 4, based on the Surface Under the Cumulative Ranking Curve (SUCRA) values, protein supplementation ranked highest for improving muscle strength in healthy older adults (SUCRA: 98.7%), followed by creatine (SUCRA: 48.9%), placebo (SUCRA: 43.8%), and HMB (SUCRA: 8.7%). For muscle mass, creatine supplementation ranked highest (SUCRA: 99.9%), followed by protein (SUCRA: 62.5%), HMB (SUCRA: 23.9%), and placebo (SUCRA: 13.7%).

#### 3.4.4 Meta-analysis results

The network meta-analysis (Tables 2, 3) demonstrated that, compared to resistance training alone (SUCRA: 43.8%), *protein supplementation* significantly improved muscle strength (SMD = 0.45, 95%CI: 0.20 to 0.69; SUCRA: 98.7%). *Creatine* (MD = 2.18, 95% CI: 0.93 to 3.44; SUCRA: 99.9%) and *protein* (MD = 0.37, 95% CI: 0.04 to 0.70; SUCRA: 62.5%) significantly enhanced muscle mass.

**Table 2:** SUCRA-based ranking of nutritional interventions for muscle strength.

Protein			
0.41 (−0.04,0.87)	Creatine		
0.45 (0.20,0.69)	0.03 (−0.35,0.42)	Placebo	
0.67 (0.25,1.09)	0.26 (−0.26,0.77)	0.22 (−0.12,0.57)	HMB

**Table 3:** SUCRA-based ranking of nutritional interventions for muscle mass.

Creatine			
1.81 (0.51,3.11)	Protein		
2.13 (0.81,3.45)	0.32 (−0.19,0.83)	HMB	
2.18 (0.92,3.44)	0.37 (0.04,0.70)	0.05 (−0.33,0.44)	Placebo

Pairwise comparisons revealed that protein supplementation was superior to HMB for improving muscle strength (SMD = −0.67, 95% CI: −1.09 to −0.25; SUCRA: 8.7%). Creatine supplementation outperformed both protein (MD = 1.81, 95% CI: 0.51 to 3.11) and HMB (MD = −2.13, 95% CI: −3.45 to −0.81; SUCRA: 23.9%) in increasing muscle mass.

#### 3.4.5 Publication bias

**Figure 5:**
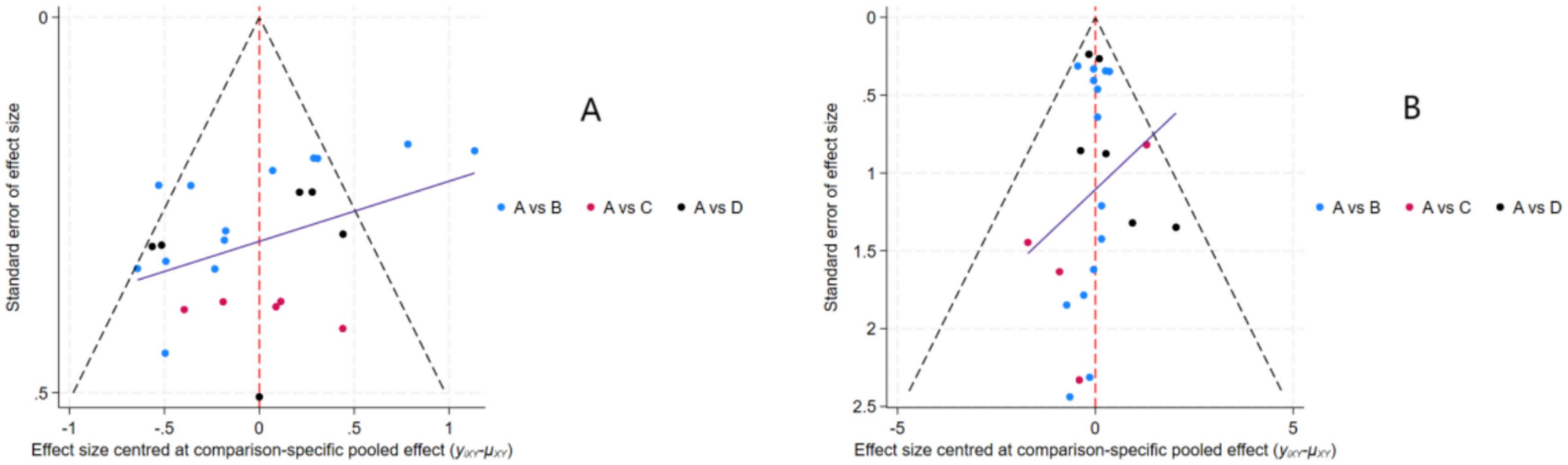
Funnel plots assessing publication bias and small-study effects: **(A)** muscle strength outcomes; **(B)** muscle mass outcomes. Intervention nodes: A = Placebo; B = Protein; C = Creatine; D = HMB.

Funnel plots assessing the effects of nutritional interventions on muscle strength and mass in healthy older adults are shown in Figures 5A,B. Both funnel plots exhibited approximate symmetry, suggesting well-distributed studies and a low likelihood of publication bias.

## 4 Discussion

This study represents the first network meta-analysis (NMA) to compare the efficacy of protein, creatine, and β-hydroxy-β-methylbutyrate (HMB) supplementation combined with resistance training in improving muscle strength and mass among healthy older adults. The findings demonstrate that protein supplementation significantly enhances both muscle strength (SMD = 0.45, 95%CI: 0.20,0.69) and mass (MD = 0.37, 95%CI: 0.04,0.70), ranked second only to creatine in terms of muscle mass improvement. (MD = 1.81, 95%CI: 0.51,3.11). Creatine supplementation yielded the most pronounced effects on muscle mass (MD = 2.18, 95%CI: 0.92,3.44; SUCRA = 99.9%), surpassing both protein and HMB. In contrast, HMB supplementation demonstrated no statistically significant effects on either muscle strength (SMD = −0.22, 95%CI: −0.57,0.12) or muscle mass outcomes (MD = 0.05, 95%CI: −0.33,0.44).

### 4.1 Protein supplementation

The superior efficacy of protein supplementation aligns with its critical role in counteracting age-related anabolic resistance, a hallmark of sarcopenia pathogenesis. Older adults require higher-quality protein intake (≥1.2 g/kg/day) to stimulate muscle protein synthesis (MPS) when combined with resistance training (21). The dosage range in this analysis aligns with Morton et al.’s (9) threshold for optimizing muscle adaptation, likely explaining the observed benefits.

For instance, Angela et al. reported a 1.8 kg increase in lean mass following 12 weeks of 40 g/day whey protein supplementation, while Hiroyasu et al. achieved a 1.2 kg gain with 1.2 g/kg/day over 24 weeks. These findings corroborate Liao et al.’s (22) meta-analysis, which synthesized 12 RCTs and concluded that 30–45 g/day of whey protein combined with ≥8 weeks of resistance training increases lean mass by 1.4 kg in older adults. Notably, Robin et al. (41) demonstrated equivalent efficacy using whole-food protein sources (220 g red meat, 6 servings/week) (23), supporting a “food-first” nutritional strategy. However, age-related declines in mastication capacity may limit the practicality of whole-food approaches in this population (24).

### 4.2 Creatine supplementation

Creatine supplementation combined with resistance training demonstrated a significant improvement in muscle mass (MD = 2.18) in healthy older adults, with an effect size 5.9 times greater than that of protein. Its absolute superiority in SUCRA rankings (99.9%) underscores its efficacy. These findings align with creatine’s unique role in cellular energy metabolism. By increasing phosphocreatine reserves, creatine facilitates rapid ATP regeneration, prolongs high-intensity exercise during resistance training, and amplifies mechanical tension on muscle fibers (25). Although creatine did not show statistically significant effects on muscle strength (SMD = 0.03), its pronounced impact on muscle mass suggests that it promotes structural remodeling via myofibrillar protein accretion rather than neuromuscular adaptation (7), a mechanism well-suited to the anabolic characteristics of aging muscle. Creatine enhances muscle hypertrophy through two synergistic pathways, Direct osmotic effects: Increased intramyocellular creatine concentrations elevate osmotic pressure, stimulating cellular hydration and activating protein synthesis signaling pathways (26).

Indirect mechanical overload: Improved training capacity enhances mechano-growth factor release, further promoting hypertrophy (27).

In the studies included in this NMA, Andrea et al. and Chrusch et al. utilized a loading phase (20–25 g/day for 5 days) followed by maintenance dosing (5 g/day), achieving lean mass gains of 2.3–3.1 kg. These results support Candow et al.’s (28) “creatine loading threshold theory” for older adults, which posits that reduced endogenous creatine synthesis (30% lower than in younger adults) necessitates higher doses to achieve creatine pool saturation (29). The average intervention duration of 12 weeks in creatine-supplemented groups aligns with Forbes et al.’s (30) systematic review, which concluded that ≥8 weeks are required for cumulative effects on muscle mass.

### 4.3 HMB supplementation

HMB, a metabolite of leucine, theoretically functions through dual mechanisms: suppressing muscle catabolism by inhibiting the ubiquitin-proteasome system to reduce muscle breakdown (31), and activating protein synthesis via stimulation of the mTOR pathway (32). This study found that HMB supplementation combined with resistance training failed to significantly improve muscle strength (SMD = −0.22, 95%CI: −0.57, 0.12) or muscle mass (MD = 0.05, 95%CI: −0.33, 0.44) in healthy older adults, with HMB ranking lowest in SUCRA values (strength: 8.7%; muscle mass: 23.9%). These conclusions align closely with both the 2023 ABCD Supplement Classification Framework updated by the Australian Institute of Sport (AIS), which categorizes HMB as Class C evidence (33). Furthermore, a meta-analysis by Javier et al. demonstrated that for adults aged 50 to 80 years, HMB supplementation adjunctive to conventional physical exercise regimens either yielded no statistically significant effects or elicited only marginal improvements in body composition parameters, muscle strength outcomes, or physical performance metrics (34). Therefore, existing evidence does not confirm clinically significant benefits of HMB with resistance training in healthy older populations.

Despite variations in the resistance training protocols (intensity, frequency, duration) and supplement dosages among the included studies, these factors are unlikely to substantially confound the primary conclusions of the present study.

Regarding training frequency, all interventions met or exceeded the minimum threshold (≥2 sessions/week) recommended by the American College of Sports Medicine (ACSM) for older adults (5). Furthermore, Pina et al. demonstrated that resistance training (RT)-induced muscular adaptations occur in older adults regardless of whether training is performed twice or three times weekly, with both frequencies providing similar adaptations (35). Training intensity could not be quantitatively evaluated due to the lack of precise %1RM data reported in the original studies. However, all trials explicitly employed standardized resistance training, inherently ensuring therapeutic intensity ranges. Concerning intervention duration, the variation in resistance training periods (6–24 weeks) across the included studies had a limited impact on the efficacy assessment. A systematic review by Brittany et al. indicated that in untrained individuals, significant increases in muscle hypertrophy relative to baseline can be expected within the initial weeks following the commencement of training. However, this growth trajectory tends to plateau, and the rate of gain slows, around approximately 12 weeks (36).

A meta-analysis by Ryoichi et al. noted that during resistance training, the incremental benefit of protein supplementation rapidly diminishes when total protein intake exceeds 1.3 g/kg BW/d. This finding suggests that the efficiency of ingested protein conversion into lean body mass (LBM) decreases when sufficient or greater amounts of protein are consumed (37). For the different forms of HMB supplementation, the International Society of Sports Nutrition (ISSN) states that two forms of HMB are currently used: HMB-Ca and HMB-FA. HMB-FA may increase plasma absorption and retention of HMB to a greater extent than HMB-*Ca.* However, research on HMB-FA is in its early stages, and currently, insufficient evidence exists to definitively support the superiority of either form (38).

In summary, coupled with the present network meta-analysis demonstrating no statistically significant global inconsistency (muscle strength: *p* = 0.2001; muscle mass: *p* = 0.789; both > 0.05), these findings collectively indicate that the results of this study are robust.

### 4.4 Safety

Long-term safety evidence supports the sustained use of creatine and protein supplementation in older adults. A 5-year follow-up study by Gualano et al. (39) demonstrated that creatine supplementation (5 g/day) in healthy older adults did not induce renal dysfunction (ΔeGFR = −1.2 mL/min). However, caution is warranted for ultra-high protein intake (>2.0 g/kg/day), which may accelerate glomerulosclerosis; regular monitoring of urinary nitrogen excretion is recommended (40). For HMB, long-term safety data remain limited, with no systematic evidence from extended monitoring studies. Further research is required to validate its safety profile.

### 4.5 Limitations

This study has several limitations that warrant careful consideration. First, although 11 randomized controlled trials on protein supplementation were systematically identified and included, variations in protein sources and the limited number of eligible studies precluded subgroup analyses by protein type. This limitation may obscure the dose–response relationships specific to particular protein forms. Second, the evidence base for HMB interventions remains relatively limited. Only three studies with small sample sizes were included, notably the study by Din et al., which involved only 16 participants. This may lead to an underestimation of the potential effects of HMB, particularly when considering age-related differences in HMB absorption efficiency among older adults. Lastly, future investigations should prioritize larger sample sizes and incorporate newer nutritional supplements. This will enable the exploration of synergistic mechanisms between different supplements, thereby providing more refined evidence to support personalized nutrition and exercise regimens.

## 5 Conclusion

This network meta-analysis demonstrates that protein supplementation combined with resistance training significantly improves both muscle strength and mass in healthy older adults, with comparable efficacy to creatine for strength enhancement. Creatine supplementation exhibited superior efficacy for increasing muscle mass, outperforming both protein and HMB. In contrast, HMB supplementation provided no significant benefits for either outcome. To maximize the synergistic effects of nutrition and resistance training, integrated supplementation strategies prioritizing protein and creatine should be developed, with careful attention to dosage, formulation, and intervention duration. Future studies should clarify response heterogeneity across older subpopulations (e.g., sarcopenic individuals) and establish long-term safety and dose–response relationships to optimize personalized exercise-nutrition regimens.

## Data Availability

The original contributions presented in the study are included in the article/supplementary material, further inquiries can be directed to the corresponding authors.
